# Supplementary material for: Identification of photoperiod-induced specific miRNAs in the adrenal glands of Sunite sheep (Ovis aries)
Source: Front Vet Sci. 2022 Jul 22;9:888207. doi: 10.3389/fvets.2022.888207 (PMC9354845; doi:10.3389/fvets.2022.888207)
Supplement: Supplementary file 1 [file Data_Sheet_1.ZIP › Supplementary file/miRDeep2 file.zip!/miRDeep2 file/Know_Structure/SPLP42A1/oar-mir-30d.pdf]

miRBase precursor : oar-mir-30d
Total read count : 175366
oar-miR-30d read count : 174684
remaining reads : 682

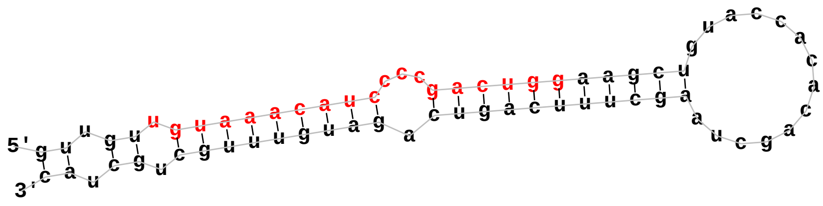

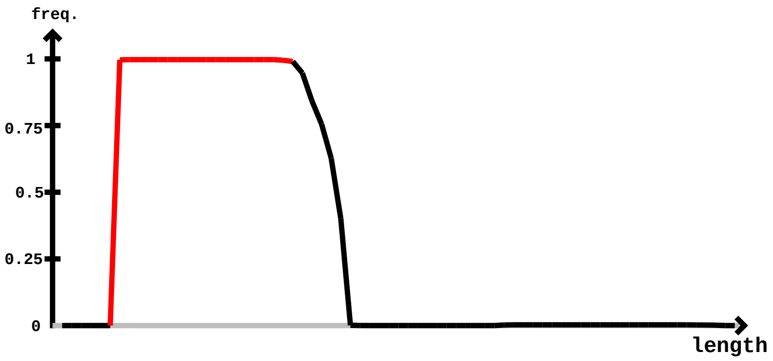


```
      oar-miR-30d
5'- guuguuguaaacauccccgacuggaagcuguaccacacagcuaagcuuucagucagauguuugcugcuac   -3'   exp
    ...((.(((((((((...(((((((((((..............))))))))))).))))))))).))...   reads   mm   sample
    ...guuguaaacauccccgacuggaagcu.........................................   1       0    seq
    ....uuguaaacauccccgacuggaagcu.........................................   2       0    seq
    .....uguaaacaucUccgacu................................................   1       1    seq
    .....uguaaacauccccgacu................................................   382     0    seq
    .....uguaaacauccccgGcu................................................   1       1    seq
    .....uguaaacaucccUgacu................................................   5       1    seq
    .....uguaaacaAccccgacu................................................   2       1    seq
    .....uguaaacauccccgacug...............................................   832     0    seq
    .....uguaaacaAccccgacug...............................................   2       1    seq
    .....uguaaacauccccgacugg..............................................   7588    0    seq
    .....uguaaacauccccgacugga.............................................   18404   0    seq
    .....uguaaacauccccgacuggaa............................................   15162   0    seq
    .....uguaaacauccccgacuggaag...........................................   22416   0    seq
    .....uguaaacauccccgacuggaagc..........................................   39748   0    seq
    .....uguaaacauccccgacuggaagcu.........................................   70025   0    seq
    .....uguaaacauccccgacuggaagcug........................................   155     0    seq
    .....uguaaacauccccgacuggaagcugu.......................................   24      0    seq
    .....uguaaacauccccgacuggaagcugua......................................   1       0    seq
    .....uguaaacauccccgacuggaagcuguac.....................................   3       0    seq
    .....uguaaacauccccgacuggaagcuguacc....................................   1       0    seq
    ......guaaacauccccgacugg..............................................   6       0    seq
    ......guaaacauccccgacugga.............................................   9       0    seq
    ......guaaacauccccgacuggaa............................................   11      0    seq
    ......guaaacauccccgacuggaag...........................................   12      0    seq
    ......guaaacauccccgacuggaagc..........................................   20      0    seq
    ......guaaacauccccgacuggaagcu.........................................   50      0    seq
    .......uaaacauccccgacuggaagc..........................................   1       0    seq
    .......uaaacauccccgacuggaagcu.........................................   3       0    seq
    .........aacauccccgacuggaagc..........................................   1       0    seq
    .............................................cuuucagucagauguuugc......   18      0    seq
    .............................................cuuucagucagauguuugcu.....   63      0    seq
    .............................................cuuucagucagauguuugcug....   52      0    seq
    .............................................cuuucagucagauguuugcugc...   78      0    seq
    .............................................cuuucagucagauguuugcugcu..   157     0    seq
    .............................................cuuucagucagauguuugcugcua.   1       0    seq
```

oar-miR-30d

```
guuguuguaaacauccccgacuggaagcuguaccacacagcuaagcuuucagucagauguuugcugcuac
.............................................cuuucagucagauguuugcugcuac    1    0    seq
..............................................uuucagucagauguuugc......    1    0    seq
..............................................uuucagucagauguuugcu.....    11   0    seq
..............................................uuucagucagauguuugcug....    6    0    seq
..............................................uuucagucagauguuugcugc...    28   0    seq
..............................................uuucagucagauguuugcugcu..    81   0    seq
..............................................uuucagucagauguuugcugcua.    1    0    seq
```
